# Supplementary material for: Evolution of Photorespiratory Glycolate Oxidase among Archaeplastida
Source: Plants (Basel). 2020 Jan 15;9(1):106. doi: 10.3390/plants9010106 (PMC7020209; doi:10.3390/plants9010106)
Supplement: Supplementary file 1 [file plants-09-00106-s001.pdf]

Supplementary material 1

Table S1. Species names and accession numbers for phylogeny. Proteins marked with a cross were used for the ancestral sequence reconstruction.

**Archeaplastida**			**Ancestral tree**
Arabidopsis GOX2	Arabidopsis thaliana	AT3G14415	x
Camelina	Camelina sativa	XP_010501901.1	x
Capsella	Capsella rubella	XP_006297960.1	x
Eutrema	Eutrema salsugineum	XP_006407078.1	x
Brassica	Brassica oleracea var. oleracea	XP_013610435.1	x
Cucurbita	Cucurbita cv.	T10242	x
Glycine	Glycine max	Glyma.08G097300.1	x
Mimulus	Mimulus gattatus	XP_012836481.1	x
Populus	Populus trichocarpa	Potri.004G065200.1	x
Zea	Zea mays	GRMZM5G853854_T04	x
Selaginella	Selaginella moellendorffii	XP_002960597.1	
Tarenaya	Tarenaya hassleriana	XP_010542340.1	x
Physcomitrella	Physcomitrella patens	Phpat.019G012100.1	x
Spirogyra	Spirogyra pratensis		x
Nitella	Nitella hyalina	HO568574, HO568574;	x
Cyanidioschyzon	Cyanidioschyzon merolae	XP_005538305.1	x
Galdieria	Galdieria sulphuraria	EME31839.1	x
Cyanophora	Cyanophora paradoxa		x
**Chlorophyta**			
Chlamydomonas	Chlamydymonas reinhardtii	Cre03.g171300.t1.2	
Chlorella	Chlorella sp. NC64A	139480	
Helicosporidium	Helicosporidium sp. ATCC 50920	KDD75237.1	
Coccomyxa	Coccomyxa sp.C-169	28454	
Volvox	Volvox carteri nagariensis	Vocar20007888m	
**Chromalveolata**			
Albugo c.	Albugo candida	CCI41131.1	
Albugo l.	Albugo laibachii Nc14	CCA27043.1	
Aphanomyces i.	Aphanomyces invadans	XP_008863753.1	
Aphanomyces a.	Aphanomyces astaci	XP_009824694.1	
Saprolegnia p.	Saprolegnia parasitica CBS 223.65	XP_012195384.1	
Saprolegnia d.	Saprolegnia diclina VS20	XP_008607053.1	
Phytophthora s.	Phytophthora sojae	XP_009537350.1	
Phytophthora P1569	Phytophthora parasitica P1569	ETI31031.1	
Phytophthora INRA-310	Phytophthora parasitica INRA-310	XP_008915124.1	
Phytophthora CJ01A1	Phytophthora parasitica CJ01A1	ETP00827.1	
Phytophthora i.	Phytophthora infestans T30-4	XP_002904015.1	
Ichthyophthirius	Ichthyophthirius multifiliis	XP_004030568.1	

Tetrahymena	Tetrahymena thermophila SB210	XP_001018684.1	
Paramecium	Paramecium tetraurelia strain d4-2	XP_001450828.1	
Oxytricha	Oxytricha trifallax	EJY79775.1	
Stylonychia	Stylonychia lemnae	CDW90415.1	
Nannochloropsis	Nannochloropsis gaditana	EWM20821.1	
Laminaria	Laminaria digitata	CAB61335.1	
Ectocarpus	Ectocarpus siliculosus	CBJ25884.1	
**Metazoa**			
Anolis	Anolis carolinensis	XP_003215389.1	
Gallus	Gallus gallus	NP_001186371.2	
Felis	Felis catus	XP_003983819.1	
Dipodomys	Dipodomys ordii	XP_012866539.1	
Ursus	Ursus maritimus	XP_008701559.1	
Pongo	Pongo abelii	XP_002829994.1	
Homo	Homo sapiens	ENST00000361035	
Latimera	Latimera chalumnae	XP_006007815.1	
Rattus	Rattus norvegicus	ENSRNOT0000000 6330	
Saccoglossus	Saccoglossus kowalevskii	XP_006813604.1	
Tribolium	Tribolium castaneum	XP_970519.1	
**Cyanobacteria**			
Nostoc 7524	Nostoc sp. PCC 7524	WP_015140379.1	x
Cylindrospermum	Cylindrospermum stagnale	WP_015205993.1	x
Anabaena 7108	Anabaena sp. PCC 7108	WP_016953564.1	x
Tolypothrix	Tolypothrix sp. PCC 7601	WP_045868031.1	x
Anabaena 90	Anabaena sp. 90	WP_015080525.1	x
Nostoc	Nostoc sp. PCC 7120	all0170	x
Calothrix	Calothrix sp. PCC 7507	WP_015130234.1	x
Trichodesmium	Trichodesmium erythraeum	WP_011611983.1	x
Microcoleus	Microcoleus sp. PCC 7113	WP_015184688.1	x
Scytonema	Scytonema hofmanni	WP_017741674.1	x
Fischerella	Fischerella muscicola	WP_016861129.1	x
Mastigocoleus	Mastigocoleus testarum	WP_027846885.1	x
Chlorogloeopsis	Chlorogloeopsis fritschii	WP_016877915.1	x
Anabaena	Anabaena variabilis	WP_011318254.1	x
**Actinobacteria and Nonsulfur Bacteria**			
Streptomyces 11814	Streptomyces albulus CCRC 11814	WP_044371981.1	x
Streptomyces au.	Streptomyces auratus	WP_006606736	x
Kitasatospora	Kitasatospora sp. NRRL B-11411	WP_030458771.1	x
Streptomyces a.	Streptomyces albulus	WP_016579310	x
Streptomyces 337	Streptomyces sp. NRRL S-337	WP_051798467.1	x
Streptomyces 1448	Streptomyces sp. NRRL S-1448	WP_037807924.1	x
Herpetosiphon	Herpetosiphon aurantiacus DSM 785	ABX04514.1	
Ktedonobacter	Ktedonobacter racemifer	WP_007918812	

Thermogemmatispora	Thermogemmatispora carboxidivorans	WP_052888153.1
**Verrucomicrobia and Proteobacteria**		
Verrucomicrobium	Verrucomicrobium spinosum	WP_009963469.1
Verrucomicrobium BvORR034	Verrucomicrobium sp. BvORR034	WP_050027044.1
Verrucomicrobium BvORR106	Verrucomicrobium sp. BvORR106	WP_038170772.1
Haloferula	Haloferula sp. BvORR071	WP_035603332.1
Acetobacteraceae	Acetobacteraceae bacterium AT-5844	WP_007438300.1
Bosea	Bosea sp. 117	WP_029352590.1
Roseomonas	Roseomonas gilardii	WP_051417580.1
Opitutaceae	Opitutaceae bacterium TAV1	WP_007361734.1
Azospira	Azospira oryzae	WP_014236754.1
Methylocystis	Methylocystis parvus	WP_026016490.1
Azotobacter c.	Azotobacter chroococcum	WP_039805662.1
Azotobacter v.	Azotobacter vinelandii DJ	WP_012699882.1
Novosphingobium	Novosphingobium pentaromativorans US6-1	WP_041565053.1
Pusillimonas	Pusillimonas noertemannii	WP_040548591.1
Alcaligenes	Alcaligenes faecalis	WP_042482826.1
Pseudomonas sp.	Pseudomonas sp. LFM046	WP_044872130.1
Pseudomonas r.	Pseudomonas resinovorans	WP_028628539.1
Pseudomonas p.	Pseudomonas pseudoalcaligenes KF707	WP_003452025.1
Pseudomonas a.	Pseudomonas alcaligenes	WP_043244200.1
Parvibaculum	Parvibaculum lavamentivorans	WP_011995292.1
Polaromonas	Polaromonas naphthalenivorans	WP_049763759.1
Advenella	Advenella mimigardefordensis DPN7	WP_025373200.1
Hydrogenophaga	Hydrogenophaga sp. PBC	EIK91695.1
**Firmicutes and Euryarchaeota**		
Halovivax a.	Halovivax asiaticus	WP_007699127.1
Halovivax r.	Halovivax ruber	WP_015299795.1
Halobiforma	Halobiforma lacisalsi	WP_007142116.1
Natrialba	Natrialba hulunbeirensis	WP_006653595.1
Haladaptatus	Haladaptatus cibarius	WP_049971073.1
Halopiger	Halopiger salifodinae	WP_049989561.1
Acidaminococcus D21	Acidaminococcus sp. D21	EEH91403
Aerococcus	Aerococcus viridans	AAB36100
Enterococcus 1	Enterococcus faecium	WP_002296473
Enterococcus 2	Enterococcus faecium	WP_047929821.1
Enterococcus 3	Enterococcus pallens ATCC BAA-351	WP_010759649.1
Lactococcus	Lactococcus garvieae	WP_003134622.1

Table S2: Catalytic efficiency of recombinant GOX and GOX-like proteins.

Enzyme	Organism	Reference	K_{cat}/K_M (s^{-1} mol^{-1} l)	
			L-Lactate	Glycolate
N3-GOX	Synthetic ancestral protein	This study	78	467
No-LOX[x]	*Nostoc* sp. PCC 7120	Hackenberg *et al.*[x]	2068707	1413
Cr-LOX[x]	*Chlamydomonas reinhardtii*	Hackenberg *et al.*[x]	904571	1047
Cp-GOXc	*Cyanophora paradoxa*	This study	5240	165418
Cm-GOX[y]	*Cyanidioschyzon merolae*	Rademacher *et al.*[y]	1534	12444
Sp-GOX	*Spirogyra pratensis*	This study	10399	199220
At-GOX2[x]	*Arabidopsis thaliana*	Hackenberg *et al.*[x]	13703	124398

[x] [30]; [y] [38]

Table S3: Sequences of the used primer sequences.

Primer	Sequence	Restriction enzyme
CpGOXb_275_fw<br>CpGOXb_369_fw<br>CpGOXb_655_rv	TGGCCCCGACGGCGATGCAG<br>CTCGTGGTCGACCACCCCGC<br>ACGCGACGTACGCGGCGAGG	none
CpGOX_RV_5P<br>CpGOX_FW_5P	GACGAACCAGACGTTCGGTACATGCACGATC<br>GTGCACAGGCAGCAGGTTATAAAGCACTGTGT	none
Spra-GOX-EcoRI-fw<br>Spra-GOX-XhoI-rv	GAATTCATGTCTTCAGAAGTCGTTA<br>CTCGAGTTAAAGACGAGCGGCCATT	*Eco*RI<br>*Xho*I

Supplementary Figures

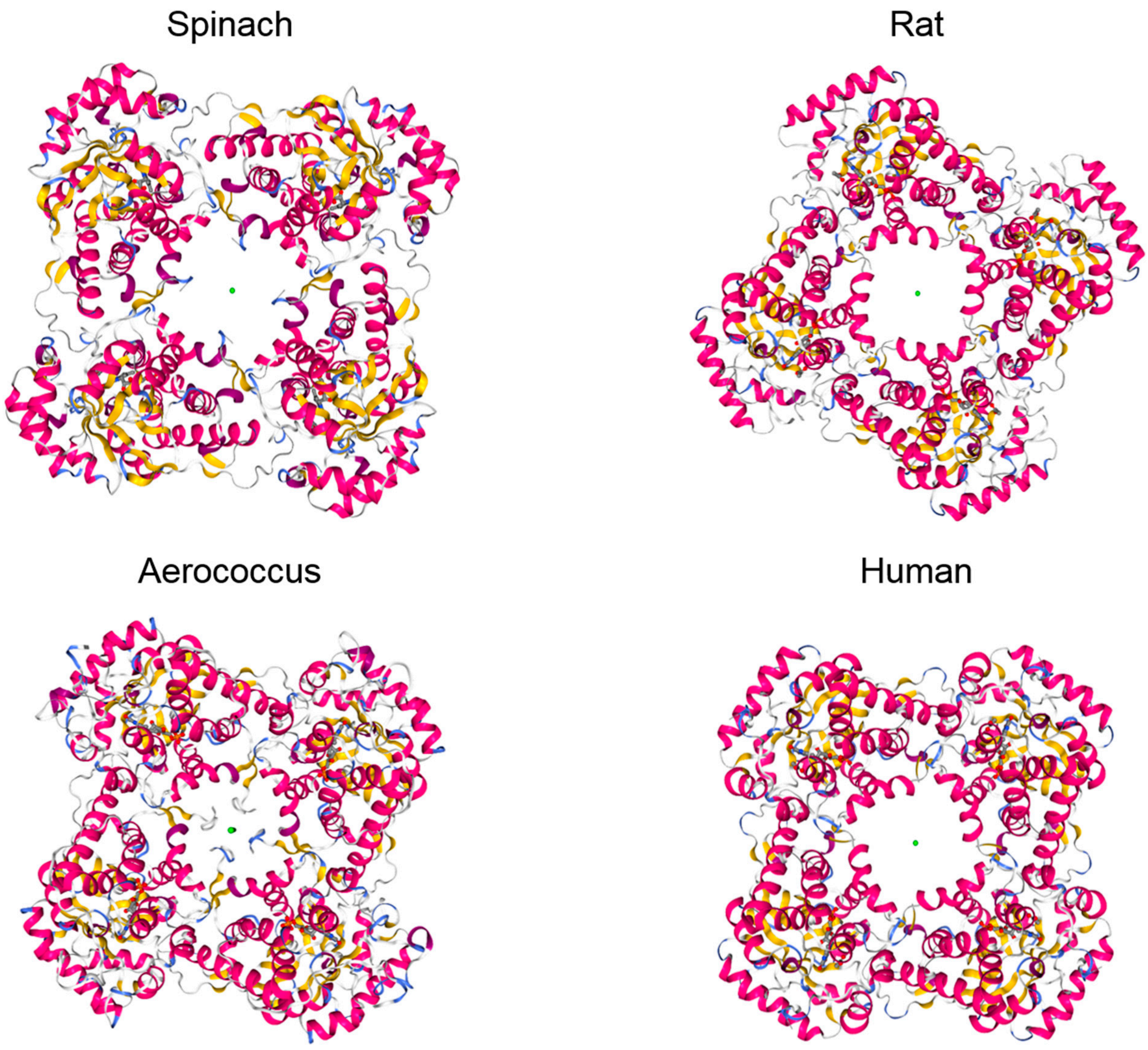

Figure S1. GOX-like proteins from eukaryotes and prokaryotes with different substrate preferences share a common tertiary and quaternary structure. Crystal structure of glycolate oxidase from spinach (1AL8) [28], long-chain hydroxyacid oxidase from rat (1TB39 [78] L-lactate oxidase from *Aerococcus* (2DU2) [79], and glycolate oxidase from human (2NZL). All proteins share the same quaternary structure build out of four identical subunits. Each subunit bound one FMN molecule. Protein structures were visualized using NGL-Viewer [80].

>Sp-GOX
ATGTCTTCAGAAGTCGTTAATGTCACAGAATTTCAGGCGATCGCCAAACAGAAATTGCCCAAGCAGACTTATGATTATTATG
CTTCTGGTGCTGAAGACGAATGGTCTCTTCGAGAAAACAGAAACGCATTTGAACGCATAACATTTCGTCCTAGAATCTTGAT
CGATGTAAGCAAAATAGATCTTACTACTTCAGTCTTAGGTTATCGACTTTCCATGCCCATCATGGTTGCTCCTACTGCAATGC
AAAAAATGGCTCATCCCGATGGTGAACTTGCTACTGCTCGTGCTGCCACTTCCATGAACACAGCCATGACCCTATCTTCCTG
GGCTACTAGCAGCGTCGAAGAAGTGTCCAGCACTGGTCCAGGCCTGAGATTCTTCCAACTATATGTGTATAAGAACAGAGA
CGTAGTCTTACAGTTAGTGAGGAGAGCAGAGAAGGCAGGATTCAAAGCTATCGCCCTCACCGTCGATACTCCTAGACTAGG
CAGGAGAGAGGCGGACATCAGAAACAAATTTGTTTTACCCCCTGGGTTGACTTTGAAAAATTTTGAAGGATTGGATTTGGGG
AAAATGGATCAGACTGAAGACTCGGGATTAGCATCCTACGTGGCAGGTCAAATCGACCGATCGCTCAGTTGGAAAGATGTA
AAATGGCTTCAGAGCGTCACAAGCATGCCTATTTTGGTGAAGGGAGTTATCACTGGAGAAGATGCCAAGATTGCAGTAGAA
TGCGGCGCATCTGGCATCATCGTGTCCAATCATGGAGCAAGGCAATTGGATTACGTGTCATCTACCATCAATGCATTGGAG
GAAGTTGTGAAAGCGACAGCTGGCAGGATTCCTGTGTTTTTGGATGGGGGAGTGAGGAGAGGAACGGACGTACTCAAAGC
CTTAGCTTTGGGAGCCAAGGGAGTATTCGTGGGGAGACCTGTCGTATTCGCTCTCGCTTCAGATGGAGAAGCAGGAGTGA
AGAAGTGTTTGCAAATGTTGAGGGACGAGTTCGAACTGGCCATGGCTTTAGCAGGATGCACTAAAATCTCCGACATCAACA
GAAGCTTGGTGCAGACGGAAGGAGACAGAATAAGAATGGCCGCTCGTCTTTAA

>Sp-GOX
MSSEVVNVTEFQAIAKQKLPKQTYDYYASGAEDEWSLRENRNAFERITFRPRILIDVSKIDLTTSVLGYRLSMPIMVAPTAMQKMA
HPDGELATARAATSMNTAMTLSSWATSSVEEVSSTGPGLRFFQLYVYKNRDVVLQLVRRAEKAGFKAIALTVDTPRLGRREADIR
NKFVLPPGLTLKNFEGLDLGKMDQTEDSGLASYVAGQIDRSLSWKDVKWLQSVTSMPILVKGVITGEDAKIAVECGASGIIVSNHG
ARQLDYVSSTINALEEVVKATAGRIPVFLDGGVRRGTDVLKALALGAKGVFVGRPVVFALASDGEAGVKKCLQMLRDEFELAMAL
AGCTKISDINRSLVQTEGDRIRMAARL*

Figure S2. Sequence of the cDNA and protein of the GOX from *Spirogyra pratensis* used in this study.

>gCp-GOX
ATGCAGCCGCTGAGCGCGCAAGCGCTAGCCCAATACCGCGCCGCAGCGCCGATGGACCCCGTGAACGTGATGGAGTACC
AGATGGTGGCGAAGGAGAAGCTGCCGAAGATGGTGTACGATTACTACGCGTCGGGCGCCGACGACCAGTGGACTCTGCA
GGAGGCGCGCTCGGCTTTCGAGCGGATCACCTTCCGGCCGCGCGTGCTCGTGGACGTCTCCGTTCAGGATATGAGCACC
ACCATCCTTGGCCAGAAGGTCGCGTTCCCGATCTGCGTGGCCCCGACGGCGATGCAGTGCATGGCCACCGGCCAGGGCG
AGAAGGCGACCTCGCGCGCCGTGGCCTCGATGAACACCCTCATGACCCTTAGCTCGTGGTCGACCACCCCGCTCGAGGA
GGTCGCCAAGGCGGCCCCGCACGGCGCGCGCTGGTTCCAGCTCTACGTCTACAAGGACCGCGCATGCACCGAGCGCCTC
GTCCGCCGCGCCCAGGCCGCCGGCTACAAGGCTCTCTGCCTCACGGTCGACACCCCCCGCCTCGGCCGCCGTGAGAAC
GACGTGAAGAACCGCTTCCACCTGCCGGACGGCCTCACCATGGGCAACTTCCTCGACCTCGTCCAGGCCGAGATGCCGA
CTGACCAGAAGGACTCGGGCCTCGCCGCGTACGTCGCGTCCCTCATCGACCCCACCCTCAACTGGAAGGACGTCGAGTG
GCTCAAGACCATCACCAACCTGCCGATTGTCGTGAAGGGCGTGCTGACGGCGGAGGACGCGCTGATGGCGGTGAAGCAC
GGCTGCGCCGGCATCGTCGTGTCGAACCACGGCGCGCGGCAGCTCGACTACAGCCCGGCGACCATCGACGTGCTCGAG
GAGGTCGTCAACGCGGTCCGCGGCTACCCGATCGAGGTCTACCTCGACGGGGGCGTCCGGAGGGGCACCGACGCCCTC
AAGGCCCTCGCCCTCGGCGCCCGCGCGGTCTTCGTGGGGCGGCCGGTGGTGTGGGGCCTCGCGGCGGGCGGCGAGGA
GGGCGTGCGCAAGGTGCTGACGATGCTGCGGGACGAGTTCAAGCTCGGCCTCGCGCTGCTCGGCTGCCAGTCCCTCAAG
GACATCAACCGCTCGATGGTCGTCACCCCCGCCGACCGCGCCAAGACCGCCGTCCGCGCCCGCCTCTGA

>Cp-GOX
ATGCAGCCGCTGAGCGCACAGGCACTGGCACAGTATCGTGCAGCAGCACCGATGGATCCGGTTAATGTTATGGAATATCA
GATGGTGGCCAAAGAAAAACTGCCGAAAATGGTGTATGATTATTATGCAAGCGGTGCAGATGATCAGTGGACCCTGCAAGA
AGCACGTAGCGCATTTGAACGTATTACCTTTCGTCCGCGTGTTCTGGTTGATGTTAGCGTTCAGGATATGAGCACCACCATT
CTGGGTCAGAAAGTTGCATTTCCGATTTGTGTTGCACCGACCGCAATGCAGTGTATGGCAACCGGTCAGGGTGAAAAAGCA
ACCAGCCGTGCAGTTGCAAGCATGAATACCCTGATGACCCTGAGCAGCTGGTCAACCACACCGCTGGAAGAAGTTGCAAA
AGCCGCACCGCATGGTGCACGTTGGTTTCAGCTGTATGTTTATAAAGATCGTGCATGTACCGAACGTCTGGTTCGTCGTGC
ACAGGCAGCAGGTTATAAAGCACTGTGTCTGACCGTTGATACACCGCGTCTGGGTCGTCGTGAAAATGATGTTAAAAATCG
TTTTCATCTGCCGGATGGTCTGACCATGGGTAATTTTCTGGATCTGGTTCAGGCAGAAATGCCGACCGATCAGAAAGATAGT
GGCCTGGCAGCCTATGTTGCAAGCCTGATTGATCCGACCCTGAATTGGAAAGATGTTGAATGGCTGAAAACCATTACCAAT
CTGCCGATTGTTGTTAAAGGTGTTCTGACAGCCGAAGATGCACTGATGGCAGTTAAACATGGTTGTGCAGGTATTGTTGTGA
GCAATCATGGTGCCCGTCAGCTGGATTATAGTCCGGCAACCATTGATGTTCTGGAAGAGGTTGTTAATGCAGTTCGTGGTT
ATCCGATTGAAGTTTATCTGGATGGTGGTGTTCGTCGTGGCACCGATGCCCTGAAAGCACTGGCCCTGGGTGCACGTGCC
GTTTTTGTTGGTCGTCCGGTTGTTTGGGGACTGGCAGCCGGTGGTGAAGAAGGTGTTCGTAAAGTTCTGACAATGCTGCGT
GATGAATTTAAACTGGGTCTGGCACTGCTGGGTTGTCAGAGCCTGAAAGATATCAATCGTAGCATGGTTGTTACACCGGCA
GATCGTGCCAAAACCGCAGTGCGTGCCCGTCTGTAA

>Cp-GOX
MQPLSAQALAQYRAAAPMDPVNVMEYQMVAKEKLPKMVYDYYASGADDQWTLQEARSAFERITFRPRVLVDVSVQDMSTTILG
QKVAFPICVAPTAMQCMATGQGEKATSRAVASMNTLMTLSSWSTTPLEEVAKAAPHGARWFQLYVYKDRACTERLVRRAQAA
GYKALCLTVDTPRLGRRENDVKNRFHLPDGLTMGNFLDLVQAEMPTDQKDSGLAAYVASLIDPTLNWKDVEWLKTITNLPIVVKG
VLTAEDALMAVKHGCAGIVVSNHGARQLDYSPATIDVLEEVVNAVRGYPIEVYLDGGVRRGTDALKALALGARAVFVGRPVVWG
LAAGGEEGVRKVLTMLRDEFKLGLALLGCQSLKDINRSMVVTPADRAKTAVRARL*

Figure S3. Corrected sequence of the Cp-GOX coding gene in *Cyanophora paradoxa* (the underlined nucleotides represent the miss-annotated intron; upper panel), the codon-optimized cDNA and protein used in this study. The codon usage was optimized referring to the used overexpressing system *E. coli.*

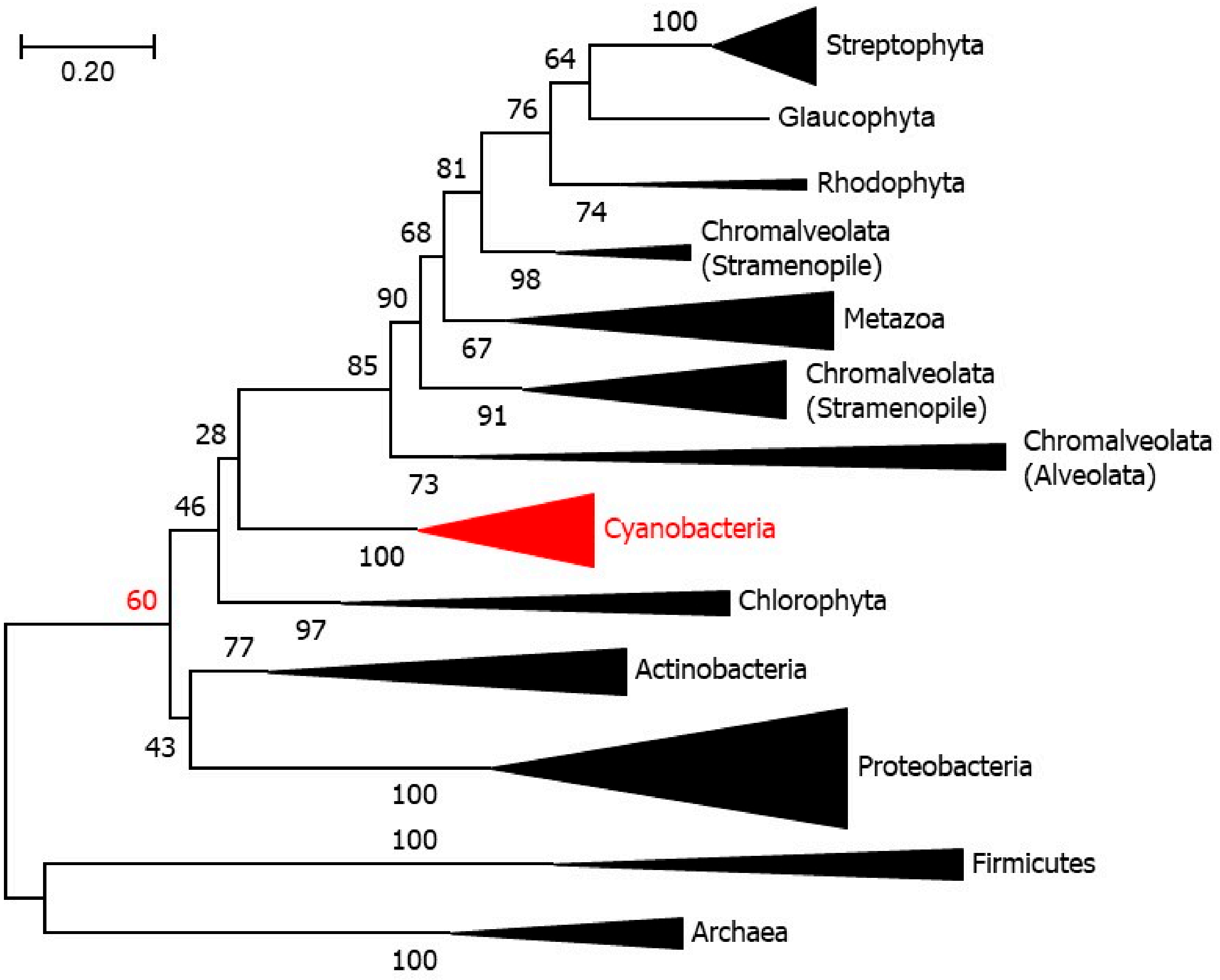

Figure S4. Collapsed phylogenetic tree of GOX-like proteins based on 111 amino acid sequences from all organismic groups in the tree of life. The phylogeny was implemented using Maximum likelihood and the LG substitution model assuming an estimated proportion of invariant sites and a gamma distribution. Bootstrap values were inferred from 1000 replicates. The species names and accession numbers of GOX-like proteins are listed in the Supplementary Material 1, table S1.

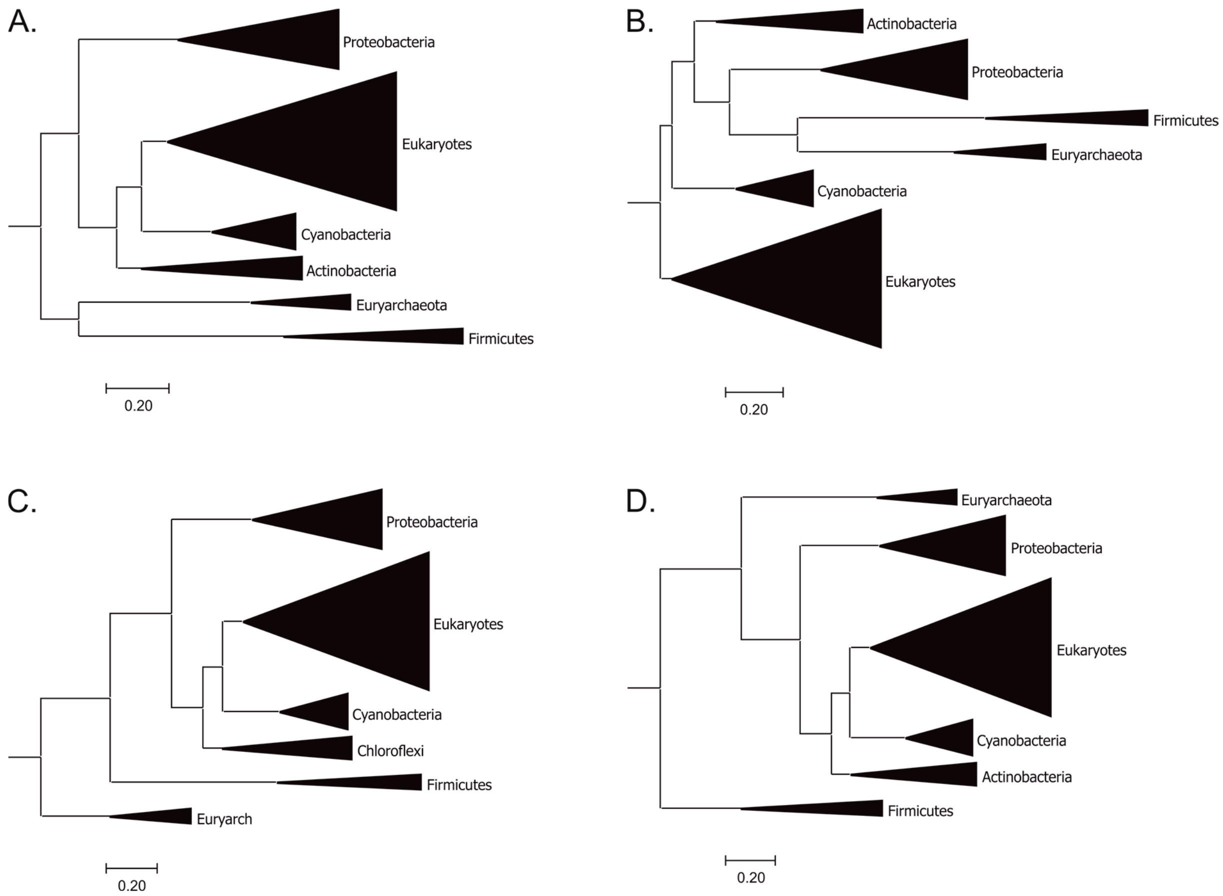

Figure S5. Alternative rooting of phylogenetic trees of GOX- and LOX-like sequences. For details see Figure 2. (A) Midpoint rooted tree. The root is placed between the branch of Euryarchaeota and Firmicutes and the remaining clusters (as in Figure 1). (B) The root is placed between Eukaryotes and the other clusters (as in [32]). (C) The root is placed between the branch of Archaea and the other clusters. (D) The root is placed between the branch of Firmicutes and the other clusters.

>N3-GOX
MNVMEYQALAKEKLPKMVYDYYASGADDQWTLQENRSAFERIRFRPRVLVDVSKQDLSTTILGQPVSFPIMVAPTAMQCMAHP
EGELATARAAASAGTIMTLSSWSTTSLEDVAAAAGPRWFQLYVYKDRAVTERLVRRAEAAGYRALCLTVDTPRLGRREADIRNR
FHLPPGLTLANFADLALEMPEQQGDSGLAAYVASLIDPSLSWKDVEWLKSITSLPIVVKGVLTAEDAQMAVQHGAAGIIVSNHGAR
QLDYAPATIDALEEVVKAVRGRVEVFLDGGVRRGTDVLKALALGARAVFVGRPVVWGLAVGGEEGVRKVLSMLRDEFELAMAL
CGCQKLSDINRSMVL*

>N3-GOX
ATGAACGTTATGGAATATCAGGCACTGGCAAAAGAAAAACTGCCGAAAATGGTGTATGATTATTATGCAAGCGGTGCAGATG
ATCAGTGGACCCTGCAAGAAAATCGTAGCGCATTTGAACGTATTCGTTTTCGTCCGCGTGTTCTGGTTGATGTTAGCAAACA
GGATCTGAGCACCACCATTCTGGGTCAGCCGGTTAGCTTTCCGATTATGGTTGCACCGACCGCAATGCAGTGTATGGCACA
TCCGGAAGGTGAACTGGCAACCGCACGTGCAGCAGCAAGCGCAGGCACCATTATGACCCTGAGCAGCTGGTCAACCACCA
GTCTGGAAGATGTTGCAGCAGCCGCAGGTCCGCGTTGGTTTCAGCTGTATGTTTATAAAGATCGTGCAGTTACCGAACGTC
TGGTTCGTCGTGCCGAAGCAGCAGGTTATCGTGCACTGTGTCTGACCGTTGATACACCGCGTCTGGGTCGCCGTGAAGCA
GATATTCGTAATCGTTTTCATCTGCCTCCGGGTCTGACCCTGGCAAATTTTGCAGATCTGGCACTGGAAATGCCGGAACAG
CAGGGTGATAGCGGTCTGGCAGCATATGTTGCAAGCCTGATTGATCCGAGCCTGAGCTGGAAAGATGTGGAATGGCTGAA
AAGCATTACCAGCCTGCCGATTGTTGTTAAAGGTGTTCTGACAGCCGAAGATGCACAGATGGCAGTTCAGCATGGTGCAGC
AGGTATTATTGTTAGCAATCATGGTGCACGTCAGCTGGATTATGCACCGGCAACCATTGATGCACTGGAAGAAGTTGTGAAA
GCAGTTCGTGGTCGTGTTGAAGTTTTTCTGGATGGTGGTGTGCGTCGTGGCACCGATGTTCTGAAAGCACTGGCCCTGGG
TGCACGTGCCGTTTTTGTTGGTCGTCCGGTTGTTTGGGGTCTGGCCGTTGGTGGTGAAGAAGGTGTTCGTAAAGTTCTGAG
CATGCTGCGTGATGAATTTGAACTGGCCATGGCACTGTGTGGTTGTCAGAAACTGAGCGATATTAATCGTAGCATGGTTCT
GTAA

Figure S6. Sequence of the protein and the codon-optimized DNA of the ancestral GOX-like protein (N3-GOX) used in this study. The codon usage was optimized referring to the used overexpressing system *E. coli.*

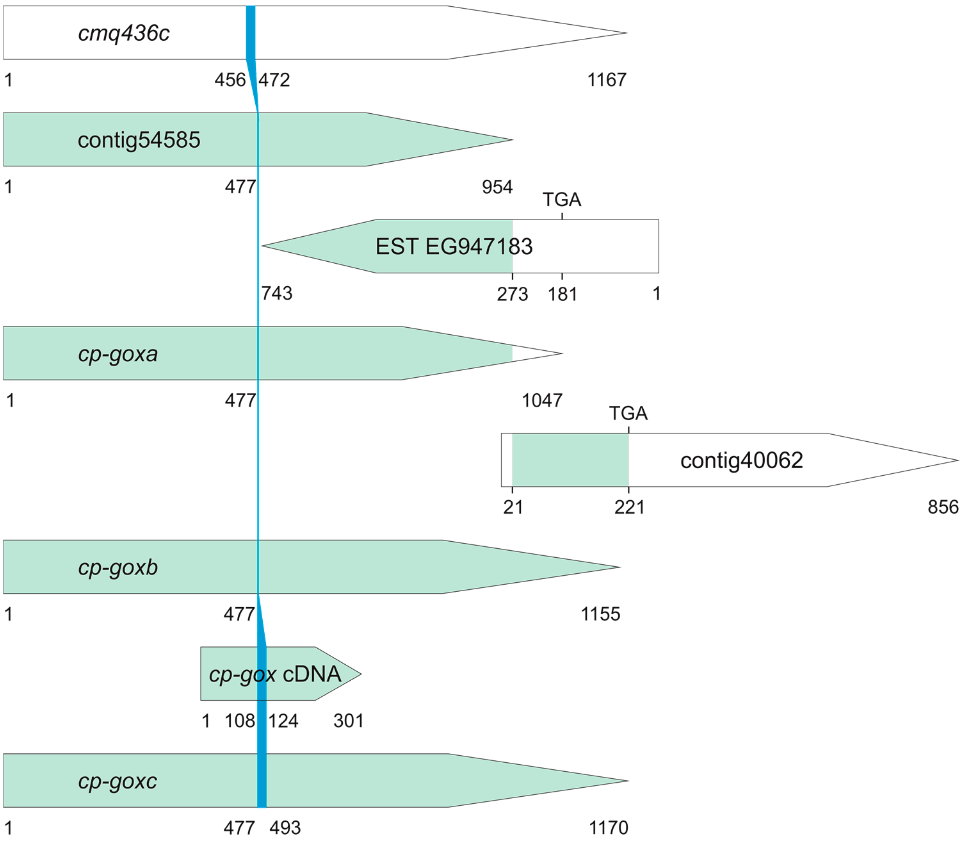

Figure S7. Schematic display of the work to obtain the correct complete *cp-goxc* gene from *Cyanophora paradoxa*. The genomic contig54585 from *Cyanophora* was assumed to encode for the Cp-GOX protein [21]. Homology analysis with the *gox* gene from *Cyaniodischyzon merolae* (*cmq436c*) revealed a missing C-terminus. Subsequent BLAST analyses with contig54585 showed high sequence similarity (~ 100 %) with an EST clone derived from *Cyanophora* cDNA (EST EG947183) containing the missing part for the C-terminus. The completed gene (*cp-goxa*) was cloned and used for production of recombinant protein, which, however, did not show enzymatic activity. A deeper look into the *Cyanophora* genome contigs identified the contig40062, which harbors a partial sequence (200 bp) showing high sequence similarity to the CMQ436C C-terminus. Subsequently, the two non-overlapping contigs were linked. The newly obtained fused gene sequence (*cp-goxb*) corresponds well with the homologues gene *cmq436c*. However, overexpressing the Cp-GOXb protein again resulted in an inactive enzyme. Further analyses amplifying cDNA from the *cp-gox* gene resulted in 301 bp fragment (*cp-gox* cDNA), which resulted in the discovery of a miss-annotation of the *gox* gene on contig54585. Congruent to the *cmq436c* gene, the newly obtained cDNA fragment shows a 15 bp insertion (blue), which seems to be crucial to encode a functional Cp-GOX protein. The 15 bp insert was included into the *cp-goxb* and the product was named *cp-goxc*. The final gene was overexpressed in *E. coli* resulting in enzymatic active Cp-GOX. Green parts show high sequence similarity with the *cmq436c* gene.

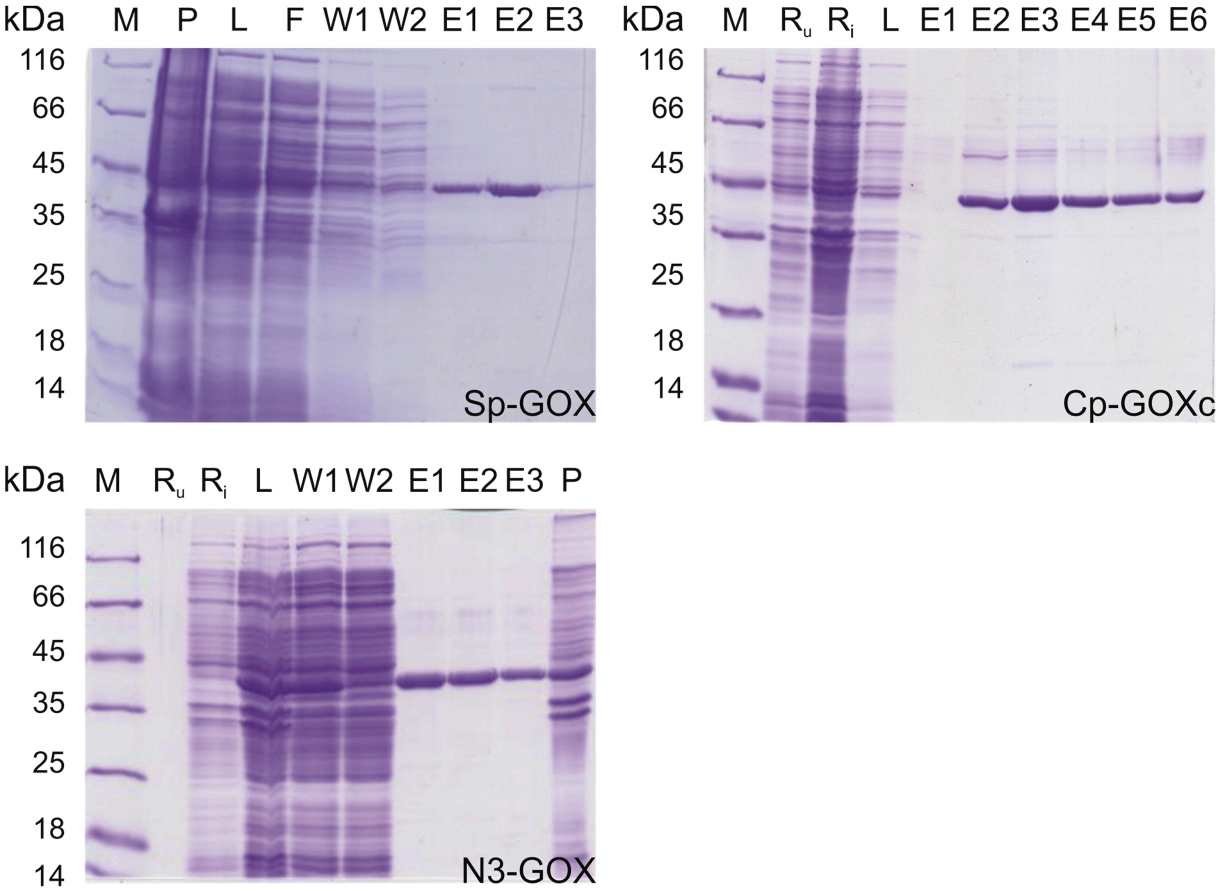

Figure S8. Purification of recombinant GOX proteins from *Spirogyra* (Sp-GOX) and *Cyanophora* (Cp-GOXc) and the ancestral GOX protein (N3-GOX). The coding sequence of the overexpressed proteins were cloned into pASG-IBA43plus and purified using the fused His- or Strep-tag. Proteins were separated by SDS-Page and stained using Coomassie brilliant blue. For enzyme assays, the elution fractions were combined and re-buffered. M, Molecular mass standard (Broad Range, Bio-Rad); P, suspended pellet after cell disruption and centrifugation; R_u, Crude extract from non-induced *E. coli* culture; R_i, Crude extract from induced *E. coli* culture; L, Cell-free protein lysate; F, Flow-through of protein extracts; W1 and W2, Washing flow-through; E1 - E6, Elution fractions.

References

78. Cunane, L.M.; Barton, J.D.; Chen, Z.-w.; Lê, K.H.D.; Amar, D.; Lederer, F.; Mathews, F.S. Crystal Structure Analysis of recombinant rat kidney long chain hydroxy acid oxidase. *Biochemistry-US* **2005**, *44*, 1521–1531.
79. Umena, Y.; Yorita, K.; Matsuoka, T.; Kita, A.; Fukui, K.; Morimoto, Y. The crystal structure of L-lactate oxidase from *Aerococcus viridans* at 2.1Å resolution reveals the mechanism of strict substrate recognition. *Biochem. Biophys. Res. Commun.* **2006**, *350*, 249–256.
80. Rose, A.S.; Hildebrand, P.W. NGL Viewer: A web application for molecular visualization. *Nucleic Acids Res.* **2015**, *43*, W576–W579.
